# 8th European Conference on Rare Diseases & Orphan Products (ECRD 2016): Edinburgh, UK. 26-28 May 2016

**DOI:** 10.1186/s13023-016-0515-y

**Published:** 2016-11-01

**Authors:** Michael Schlander, Søren Holm, Erik Nord, Jeff Richardson, Silvio Garattini, Peter Kolominsky-Rabas, Deborah Marshall, Ulf Persson, Maarten Postma, Steven Simoens, Oriol de Solà Morales, Keith Tolley, Mondher Toumi, Harry Telser, James R Bonham, Helmut Hintner, Anja Diem, Martin Laimer, Réjean Hébert, Nabarun Dasgupta, Carrie E. Pierce, Melissa Jordan, Barbara Bori, Mohanad Fors, Emilie Prazakova, Simon Day, Thomas J. Croce, Jonas Fransson, Philip Wood, Anne-Grethe Lauridsen, Joanne Higgs, Vesna Stojmirova Aleksovska, Christina Olsen, Ritchie Head, Antonio Asero, Vincenzo Papa, Christa van Kan, Loic Favennec, Silvana Venturella, Michela Salvador, Alan Krol, Stephanie J. Nielsen, Birthe B. Holm, Daniel Lewi, Patricia Durão, Heather Band, Andrea West, Marinda J. A. Hammann, Marije C. Effing-Boele, Hanka K. Dekker, Amy Hunter, Amy Simpson, Gumei Liu, Katherine Needleman, Debra Lewis, Gayatri Rao, Amy Simpson, Amy Hunter, Martin J. Whitaker, Raquel Castro

**Affiliations:** 1 Institute for Innovation & Valuation in Health Care (Innoval-HC), An der Ringkirche 4, D-65197 Wiesbaden, Germany; 2 Mannheim Medical Faculty, Institute of Public Health, University of Heidelberg, Ludolf-Krehl-Strasse 7-11, D-68167 Mannheim, Germany; 3 University of Applied Economic Sciences, Ernst-Boehe-Strasse 4, D-67059 Ludwigshafen, Germany; 4 Centre for Social Ethics and Policy, University of Manchester, Oxford Road, Manchester, M13 9PL UK; 5 School of Pharmacy, University of Oslo, Sem Sælands vei 3 Farmasibygningen, N-0371 Oslo, Norway; 6 Centre for Health Economics, Monash University, Clayton, Victoria 3800 Australia; 7 IRCCS – Istituto di Richerche Farmacologiche Mario Negri, Via Giuseppe La Masa 19, I-20156 Milan, Italy; 8 Interdisziplinäres Zentrum für Public Health (IZPH), Universitätsklinikum Erlangen, Schwabachanlage 6, D-91054 Erlangen, Germany; 9 Health Research Innovation Centre, Cumming School of Medicine, University of Calgary, 2500 University Dr. NW, Calgary, Alberta T2N 1N4 Canada; 10 The Swedish Institute for Health Economics (IHE), Box 2127, S-220 02 Lund, Sweden; 11 Department of Pharmacy, University of Groningen, Antonius Deusinglaan 1, 9713 AV Groningen, The Netherlands; 12 Department of Epidemiology, University Medical Center Groningen, University of Groningen, Hanzeplein 1, 9713 GZ Groningen, The Netherlands; 13 KU Leuven Department of Pharmaceutical and Pharmacological Sciences, Herestraat 49, B-3000 Leuven, Belgium; 14 Institut Investigació Sanitaria Pere Virgili (IISPV), Avenir 1 Ppal 1ª, E-08006 Barcelona, Spain; 15 Tolley Health Economics Ltd, 19 Bath Road, Buxton, Derbyshire SK17 6HH Great Britain; 16 UFR d’Odontologie, University Claude Bernard Lyon I, 11 rue Guillaume Paradin, F-69372 Lyon, Cedex 08 France; 17 Polynomics AG, Baslerstrasse 44, CH-4600 Olten, Switzerland; 18 Sheffield Children’s NHS Foundation Trust, Sheffield, S10 2TH UK; 19 EB House Austria, Salzburg, Austria; 20 Department of Health Evaluation, Management and Policy, School of Public Health, Université de Montréal, Québec, Canada; 21 Epidemico, Inc., 50 Milk Street, 20th floor, Boston, MA 02109 USA; 22 Novartis Pharma AG, 4002 Basel, Switzerland; 23 Clinical Trials Consulting & Training Limited, North Marston, Buckinghamshire MK18 3PL UK; 24 Head of Global Patient Advocacy, Shire plc, Dublin, Ireland; 25 Sobi – Swedish Orphan Biovitrum AB (publ.), Stockholm, Sweden; 26 European Gaucher Alliance (EGA), Dursley, Gloucestershire GL11 4ND UK; 27 Ceratium Ltd, Merseyside, CH48 8AP UK; 28 SIFI SpA, Catania, Italy; 29 PSR Group B.V., Hoofdorf, 2132HN Netherlands; 30 University of Rouen, Rouen, France; 31 RTC, Pomezia, 00040 Italy; 32 Moorfields Pharmaceuticals, London, L17NH UK; 33 Rare Diseases Denmark, Blekinge Boulevard 2, Høje Taastrup, 2630 Denmark; 34 The Cure & Action for Tay-Sachs (CATS) Foundation, London, SE12 0RW UK; 35 Batten Disease Family Association (BDFA), The Old Library, 4 Boundary Road, Farnborough, GU14 6SF UK; 36 Patient Organisation for Metabolic Diseases (VKS), 8031 GJ Zwolle, The Netherlands; 37 Genetic Alliance UK, London, N1 3QP UK; 38 Office of Orphan Products Development, Office of Special Medical Programs, U.S. Food and Drug Administration, 10903 New Hampshire Avenue, Silver Spring, MD 20993 USA; 39 Genetic Alliance UK, London, N1 3QP UK; 40 University of Sheffield, Sheffield, S10 2TN UK; 41 Diurnal Limited, Cardiff, CF14 4UJ UK; 42 European Organisation for Rare Diseases – EURORDIS, Paris, France

## Abstract

O1 The European Social Preferences Measurement (ESPM) study project: social cost value analysis, budget impact, commercial life cycle revenue management, and the economics of biopharmaceutical Research & Development (R&D)

Michael Schlander, Søren Holm, Erik Nord, Jeff Richardson, Silvio Garattini, Peter Kolominsky-Rabas, Deborah Marshall, Ulf Persson, Maarten Postma, Steven Simoens, Oriol de Solà Morales, Keith Tolley, Mondher Toumi, Harry Telser

O2 Newborn Screening: the potential and the challenges

James R Bonham

O3 Untreatable disease outcomes - how would we measure them?

Helmut Hintner, Anja Diem, Martin Laimer

O4 Taking Integrated Care Forward: Experiences from Canada to inspire service provision for people living with rare disease in Europe

Réjean Hébert

O5 Listening to the patient’s voice: social media listening for safety and benefits in rare diseases

Nabarun Dasgupta, Carrie E. Pierce, Melissa Jordan

O6 Via Opta: *Mobile apps making visually impaired patients’ lives easier*

Barbara Bori, Mohanad Fors, Emilie Prazakova

O7 A report of the IRDiRC “Small Population Clinical Trial” Task Force

Simon Day

O8 HAE patient identification and diagnosis: An innovative, ‘game changing’ collaboration

Thomas J. Croce Jr.

O9 Co-creating with the community: primary packaging & administration for people with haemophilia

Jonas Fransson, Philip Wood

O10 Go with Gaucher, taking forward the next generation. How to involve young people to create a new generation of patient advocates

Anne-Grethe Lauridsen, Joanne Higgs, Vesna Stojmirova Aleksovska

P1 ODAK – Orphan Drug for Acanthamoeba Keratitis

Christina Olsen, Ritchie Head, Antonio Asero, Vincenzo Papa, Christa van Kan, Loic Favennec, Silvana Venturella, Michela Salvador, Alan Krol

P5 Rare Navigators help people living with rare diseases to manage the social – and healthcare systems Stephanie J. Nielsen, Birthe B. Holm

P6 The eAcademy for Tay-Sachs & Sandhoff disease app

Daniel Lewi, Patricia Durão

P10 The role of a patient organisation in driving the research agenda in a rare disease

Heather Band, Andrea West

P13 Expertise for rare diseases mapped

Marinda J.A. Hammann, Marije C. Effing-Boele, Hanka K. Dekker

P14 The hidden costs of rare diseases: a feasibility study

Amy Hunter, Amy Simpson

P15 FDA’s new natural history grant program: support to build a solid foundation for development of products for rare diseases

Gumei Liu, Katherine Needleman, Debra Lewis, Gayatri Rao

P17 Understanding the wider impact of adrenal insufficiency: patient organisation involvement in the TAIN project

Amy Simpson, Amy Hunter, Martin J Whitaker

P20 Bridging the gaps between medical and social care for people living with a rare disease

Raquel Castro

## O1 The European Social Preferences Measurement (ESPM) study project: social cost value analysis, budget impact, commercial life cycle revenue management, and the economics of biopharmaceutical Research & Development (R&D)

### Michael Schlander^1,2,3^, Søren Holm^4^, Erik Nord^5^, Jeff Richardson^6^, Silvio Garattini^7^, Peter Kolominsky-Rabas^8^, Deborah Marshall^9^, Ulf Persson^10^, Maarten Postma^11^, Steven Simoens^12^, Oriol de Solà Morales^13^, Keith Tolley^14^, Mondher Toumi^15^, Harry Telser^16^

#### ^1^Institute for Innovation & Valuation in Health Care (InnoVal-HC), An der Ringkirche 4, D-65197 Wiesbaden, Germany; ^2^Mannheim Medical Faculty, Institute of Public Health, University of Heidelberg, Ludolf-Krehl-Strasse 7-11, D-68167 Mannheim, Germany; ^3^University of Applied Economic Sciences, Ernst-Boehe-Strasse 4, D-67059 Ludwigshafen, Germany; ^4^Centre for Social Ethics and Policy, University of Manchester, Oxford Road, Manchester M13 9PL, UK; ^5^School of Pharmacy; University of Oslo, Sem Sælands vei 3 Farmasibygningen, N-0371 Oslo, Norway; ^6^Centre for Health Economics, Monash University; Clayton, Victoria 3800; Australia; ^7^IRCCS – Istituto di Richerche Farmacologiche Mario Negri, Via Giuseppe La Masa 19, I-20156 Milan, Italy; ^8^Interdisziplinäres Zentrum für Public Health (IZPH); Universitätsklinikum Erlangen, Schwabachanlage 6, D-91054 Erlangen, Germany; ^9^Health Research Innovation Centre, Cumming School of Medicine, University of Calgary, 2500 University Dr. NW, Calgary, Alberta, T2N 1N4, Canada; ^10^The Swedish Institute for Health Economics (IHE), Box 2127, S-220 02 Lund, Sweden; ^11^Department of Pharmacy, University of Groningen, Antonius Deusinglaan 1 9713 AV Groningen, The Netherlands, and Department of Epidemiology, University Medical Center Groningen, University of Groningen, Hanzeplein 1 9713 GZ Groningen, The Netherlands; ^12^KU Leuven Department of Pharmaceutical and Pharmacological Sciences, Herestraat 49, B-3000 Leuven, Belgium; ^13^Institut Investigació Sanitaria Pere Virgili (IISPV), Avenir 1 Ppal 1ª; E-08006 Barcelona; ^14^Tolley Health Economics Ltd, 19 Bath Road, Buxton, Derbyshire SK17 6HH, Great Britain; ^15^UFR d'Odontologie, University Claude Bernard Lyon I; 11 rue Guillaume Paradin; F-69372 Lyon Cedex 08, France; ^16^Polynomics AG, Baslerstrasse 44, CH-4600 Olten, Switzerland

##### **Correspondence:** Michael Schlander (Michael.Schlander@innoval-hc.com; michael.schlander@medma.uni-heidelberg.de; michael.schlander@hs-lu.de) – University of Applied Economic Sciences, Ernst-Boehe-Strasse 4, D-67059 Ludwigshafen, Germany

The cost of new medicines has never been as controversial as nowadays. Health care policy makers are concerned that rising drug prices may lead to unsustainable growth of health care expenditures. The debate, however, frequently suffers from confusion of cost per unit and budget impact, i.e., the notion of “affordability.”

In their quest for value for money, policy makers have turned to systematic health technology assessments (HTAs) as a tool to aid the appraisal of the clinical, economic, social, legal and ethical implications of the adoption, and the appropriate use, of medical technologies.

HTAs of interventions for rare and ultra-rare disorders (URDs) present specific challenges. Some treatments for URDs are associated with acquisition costs exceeding 100,000€ per patient year, consequently not meeting conventional benchmarks for cost effectiveness (CE). On the other hand, the budgetary impact of orphan medicinal products (OMPs) has been limited and seems unlikely to escalate massively in the near future.

Payers have often been more concerned about the cost of adding new programs than about their CE, and many HTA agencies have created exemptions for OMPs or URDs. This corresponds to the strategy of manufacturers to maximize life cycle revenues, in light of the high fixed / low variable cost structure of the R&D-based biopharmaceutical industry. Recent estimates of the average R&D cost per new product converge in a range between US-$ 1.0 and 1.9 billion, when factoring in out-of-pocket costs, risk of failure, development times, and cost of capital. The economics of R&D may, at least in part, explain the observed inverse relationship between acquisition costs per patient and prevalence.

Exempting OMPs from CE benchmarks may have been a pragmatic work-around, but created controversy about the justification of a special status for OMPs (or URDs). The situation would be different if the focus of health economic analyses was shifted towards the social value associated with patient access to interventions. Then, opportunity cost would be represented by budgetary impact (or cumulative transfer cost).

Since societal value is not fully captured by simple aggregation of incremental health gains (weighted based on individual preferences), social norms and preferences need to be taken into account, too. Despite the increasing number of studies showing the relevance of social preferences, empirical evidence has been limited regarding their relative importance, including their interaction, and the impact of framing effects. The ESPM project has been designed to further our understanding of relevant social preferences.

**Reference**

Schlander M, Garattini, Holm S, et al. Incremental cost per quality-adjusted life year gained? The need for alternative methods to evaluate medical interventions for ultra-rare disorders. J. Comp. Eff. Res. 2014; 3 (4): 399-422.

## O2 Newborn Screening: the potential and the challenges

### James R Bonham (Jim.Bonham@sch.nhs.uk)

#### Sheffield Children’s NHS Foundation Trust, Sheffield, S10 2TH, UK

The potential offered by newborn screening to improve the lives of patients by enabling early and effective treatment is well established. Each year almost 1,000 children in the USA and Europe are identified with phenylketonuria alone as part of national programs and this has operated for approximately 50 years in many well developed countries with very significant and cost effective health benefits.

More recently the potential to expand these programs to include many more metabolic conditions utilizing tandem mass spectrometry has been adopted by many European states and notably within the USA who have taken a lead in these developments, currently screening for more than 50 disorders in all babies.

While the sensitivity of testing is high and for some conditions approaches 100 %, the positive predictive value, particularly for some disorders, is less complete and may lead to uncertainty for some families. In addition, the spectrum of disease severity has widened to include mild variants with unclear significance. This requires careful management. The potential offered by next generation sequencing performed on the original dried blood spot samples as an adjunct to biochemical testing may provide useful additional information to guide treatment or even provide the means to consider new disorders but it also raises a number of key questions: how do we select the conditions to test and who should choose, how do we provide useful and understandable pre-screening information to parents and gain meaningful consent, what do we do with genetic information such as carrier status or the identification of variants that may not have significance in childhood?

Public and patient consultation is key but may require greater societal awareness to be meaningful. The opportunities afforded by improved information, given in an accessible form, during pregnancy may help inform and stimulate this debate. In relation to whole population screening programmes it is increasingly the responsibility of health service planners, not only to evaluate new technological solutions and judge their clinical effectiveness, but also to actively seek means of public engagement to educate, inform and involve potential health users and the public in the dialogue.

## O3 Untreatable disease outcomes - how would we measure them?

### Helmut Hintner, Anja Diem, Martin Laimer

#### EB House Austria, Salzburg, Austria

##### **Correspondence:** Helmut Hintner (hhintner@icloud.com) – EB House Austria, Salzburg, Austria

To measure the benefits or harms to a patient who receives an intervention is a key feature to improve quality of care. This usually involves a strategy to define and prioritize areas for improvement; to apply appropriate (i.e. reproducible, statistically significant and GCP-compliant) measures to assess these areas; to determine a baseline reference of current practices using the selected measures and to reassess and monitor the effect of interventional improvement efforts. This process may guide and focus accountability and quality improvement programs.

In rare diseases (RD) with no existing cure, an only *small number* of affected individuals along with limited registry and generalizable research data as well as inter- and intraindividually *highly variable* course (subtypes) and occasionally early mortality, the establishment of comprehensive, reliable, validated and feasible outcome measures is particularly challenging. All this prototypically applies for Epidermolysis bullosa (EB), a group of rare, heterogenous and complex heritable blistering diseases. In order to improve the interpretation and quantification of results of (partly highly promising) interventions in EB, clinical, laboratory, microscopic and ultrastructural as well as molecular outcome measures have been proposed that should assist in appropriately reflecting the disease activity and burden and capturing both, clinician assessment as well as patient/parent perceptions: iscorEB [1], BEBS [2], EBDASI [3]. A distinct example of outcome measures in untreatable EB is reflected by clinical follow up and watchful waiting in patients with EB nevi. These moles, although clinically and dermatoscopically frighteningly resembling melanomas, usually exhibit a benign course according to our experience of more than 25 years. Finally, at the edge of a new era of curative treatment approaches, ex vivo gene therapy trials have revealed highly specific and measurable determinants of interventional success [4,5].

**References**

1. Schwieger-Briel A, Chakkittakandiyil A, Lara-Corrales I, Aujla N, Lane AT, Lucky AW, Bruckner AL, Pope E. Instrument for scoring clinical outcome of research for epidermolysis bullosa: a consensus-generated clinical research tool.Pediatr Dermatol. 2015;32(1):41-52

2. Moss C, Wong A, Davies P. The Birmingham Epidermolysis Bullosa Severity score: development and validation. Br J Dermatol. 2009;160(5):1057-65

3. Loh CC, Kim J, Su JC, Daniel BS, Venugopal SS, Rhodes LM, Intong LR, Law MG, Murrell DF. Development, reliability, and validity of a novel Epidermolysis Bullosa Disease Activity and Scarring Index (EBDASI). J Am Acad Dermatol. 2014;70(1):89-97.e1-13

4. Mavilio F, Pellegrini G, Ferrari S, Di Nunzio F, Di Iorio E, Recchia A, Maruggi G, Ferrari G, Provasi E, Bonini C, Capurro S, Conti A, Magnoni C, Giannetti A, De Luca M. Correction of junctional epidermolysis bullosa by transplantation of genetically modified epidermal stem cells. Nat Med. 2006;12(12):1397-402

5. De Rosa L, Carulli S, Cocchiarella F, Quaglino D, Enzo E, Franchini E, Giannetti A, De Santis G, Recchia A, Pellegrini G, De Luca M. Long-term stability and safety of transgenic cultured epidermal stem cells in gene therapy of junctional epidermolysis bullosa. Stem Cell Reports. 2013;2(1):1-8

## O4 Taking Integrated Care Forward: Experiences from Canada to inspire service provision for people living with rare disease in Europe

### Réjean Hébert (rejean.hebert@umontreal.ca)

#### Department of Health Evaluation, Management and Policy, School of Public Health, Université de Montréal, Québec, Canada

Several models of Integrated Service Delivery networks were experimented and tested, but most of them are designed according to a full integration model. PRISMA is the only example of a coordinated-type model to be developed and fully implemented with a process and outcome evaluation. The PRISMA model includes the following components to enhance the integration: 1) formal mechanism to manage co-operation between decision-makers and managers of all services and organizations, 2) the use of a single entry point, 3) a case management process, 4) Individualized Service Plans, 5) a unique assessment tool with a case-mix system, and 6) a computerized system for communicating between institutions and professionals.

The PRISMA model was experimentally implemented in three areas (urban, rural with or without a local hospital) in Quebec, Canada and research was carried out using both qualitative and quantitative data to evaluate its process and impact. The impact study was population-based with a quasi-experimental design on 1500 subjects over 75 years old followed up for four years. Significant impact of the prevalence and incidence of functional decline, satisfaction with services and empowerment was observed. There was a reduction in the number of Emergency Room visits and hospitalisations. The overall cost was not higher in the experimental group, even when implementation cost was included.

The PRISMA model was then implemented all over the province of Quebec from 2005 to 2015. The 70 % implementation rate threshold was reached in 2014 in all regions. Budget constraints and concomitant reforms (merging of institutions) slowed down the implementation. Many lessons were learned from this implementation: the case managers should be formally trained and accredited, structural integration by merging is not necessarily fostering functional integration, information technology development is a critical factor for integration, and appropriate funding scheme should be considered as an enabling factor for integration.

The PRISMA model has already demonstrated its efficiency and feasibility for frail older persons. It could be adapted for rare disease to improve continuity of care and integration of services.

## O5 Listening to the patient’s voice: social media listening for safety and benefits in rare diseases

### Nabarun Dasgupta, Carrie E. Pierce, Melissa Jordan

#### Epidemico, Inc., 50 Milk Street, 20^th^ floor, Boston, MA, 02109 USA

##### **Correspondence:** Nabarun Dasgupta (nabarun@epidemico.com) – Epidemico, Inc., 50 Milk Street, 20^th^ floor, Boston, MA, 02109 USA

**Background**

Patient- and caregiver-generated information online arises from a patient population that is more knowledgeable about its own treatments than ever before. Digital tools like social media and mobile apps can provide information to safety intelligence in areas where other sources fall short.

**Methods**

Challenges associated with mining unstructured data, including filtering noise and translating patient vernacular to medical terms, can be addressed through automated methodologies [1,2]. In this abstract we discuss ways in which social media listening can be beneficial for rare disease patients.

**Results**

Rare disease patients post publicly about side effects they are experiencing. For example, one patient mentioned a warning sign for serious complications: “Had constant ringing in my ears since last week. Can't work out whether it's my sinuses, or if it's [orphan-designated] tobramycin related. My levels were fine.” First, data from patient- and caregiver-generated social media can be a tool for advocacy. Online patient communities can collectively change the discussion on what matters for rare diseases. Systematically mining public conversations can amplify anecdotes into powerful and accurate representations of the patient journey. Second, travel to medical appointments, even short distances, can be a difficulty for rare disease patients. Using online electronic diaries and data mining, we can allow patients to record experiences in their own words, and send processed reports to physicians. Third, social media can allow rare disease communities to define what matters to them as outcomes for new therapies. Increasing lifespan critical, but may not be the only thing that matters to patients. Improving quality of life is often relegated to “secondary endpoints.” Social listening can help reveal and prioritize other outcomes that are important. Fourth, combining genetic or clinical data with social media may identify clinical trial eligibility more quickly.

**Conclusions**

While safety data generated from digital tools may not consist of the same elements as a traditional safety report, they nonetheless may contain valuable information that to contextualize how medical products are being used. As such, digital health information is unlikely to replace spontaneous, observational and clinical data, but may serve to augment traditional data sources. Privacy concerns for patients with rare disease may be heightened relative to more prevalent conditions. Efforts to obtain explicit permission should be made, directly from patients when feasible, and at least from forum administrators and the community more broadly. Ultimately, the processed data should be provided back to patients to empower advocacy.

**References**

1. Powell GE, Seifert HA, Reblin T, Burstein PJ, Blowers J, Menius JA, Painter JL, Thomas M, Pierce CE, Rodriguez HW, Brownstein JS, Freifeld CC, Bell HG, Dasgupta N. Social Media Listening for Routine Post-Marketing Safety Surveillance. Drug Saf. 2016 May;39(5):443-54. doi: 10.1007/s40264-015-0385-6. PMID: 26798054.

2. Freifeld CC, Brownstein JS, Menone CM, Bao W, Filice R, Kass-Hout T, Dasgupta N. Digital drug safety surveillance: monitoring pharmaceutical products in twitter. Drug Saf. 2014 May;37(5):343-50. doi: 10.1007/s40264-014-0155-x.

## O6 Via Opta: *Mobile apps making visually impaired patients’ lives easier*

### Barbara Bori, Mohanad Fors, Emilie Prazakova

#### Novartis Pharma AG, 4002 Basel, Switzerland

##### **Correspondence:** Barbara Bori (barbara.bori@novartis.com) – Novartis Pharma AG, 4002 Basel, Switzerland

**Rationale and project objective**

Via Opta Apps is a suite of 4 applications, Via Opta Daily, Via Opta Navigator, Via Opta Simulator and Via Opta Eye Life that have been specifically designed for people living with low vision to improve their quality of life.

**Background**

Novartis is committed to patients and patient engagement is crucial to help the company develop services and solutions that will improve the lives of people living with low vision and their families. The co-creation of this digital tool was identified as one critical success factor. Therefore, Novartis has developed this project in partnership with patient organisations in order to address the specific needs of patients.

**Project description and results**

The project was co-created over 2 years with input from patient organisations: 2 patient forums in collaboration with Retina International, an umbrella patient organization of 20 members representing 11 European Countries and others like ECOO, EMHF, AMDAI.

■ Via Opta Daily available in 12 languages is designed as a personal assistant to help people with low vision in their activities of daily living like colour, objects, scenes and money recognition.

■ Via Opta Navigator offers an experience that can be life changing for people living with low vision by helping them to increase their mobility and regain independence as it offers turn by turn complete navigation that was built specifically for visually impaired users.

■ Via Opta Simulator has been designed for patients, their families, healthcare professionals and caregivers as an app to provide a lens at what it is like to live with visual impairment.

■ Via Opta Eye Life which provides a real life virtual reality experience demonstrating the world from a visually impaired user perspective.

The apps can be downloaded for free from Apple iOS and Android (Google Play) stores on your mobile devices and Novartis is dedicated towards improving and updating them to continue to add value in the daily life of patients.

**Partners**

Novartis is a partner of EURORDIS and of several Patient Organisations established at Regional and Country level in Europe.

**Outcomes**

■ Interaction with Patient organisations in Advisory Boards and Forums to share and co-create future digital tools.

■ Develop solutions that will improve the lives of people living with low vision and their families.

■ Raise awareness of the disease condition thanks to an international network and alliances.

## O7 A report of the IRDiRC “Small Population Clinical Trial” Task Force

### Simon Day (simon.day@CTCT-Ltd.co.uk)

#### Clinical Trials Consulting & Training Limited, North Marston, Buckinghamshire, MK18 3PL, UK

The challenges in researching therapies in rare diseases are well recognized – amongst them are the exceptionally low disease prevalence, challenges in identifying or finding patients (let alone adequate numbers of patients), small and particularly often very heterogeneous patient populations, and limited knowledge of natural history (so we often don’t know “what would have happened if…”).

The IRDiRC Therapy Scientific Committee recommends: Encouraging, supporting and establishing early and continuous dialogue on clinical development strategies and wide evidence generation (e.g. natural history, registry, clinical trial design, clinical endpoints, surrogate endpoints, patient centred outcomes, regulatory strategy, medical practice, public health strategy) with all relevant stakeholders such as patient representatives, medical experts, researchers, scientific societies, regulators, health technology assessors, payers and sponsors when appropriate. This could be done through dedicated workshops, safe harbours where knowledge could be shared in a non-competitive manner.

Furthermore, it recommends: Encouraging, supporting and developing small population clinical trials (e.g. exploring the application of innovative methods). This is an essential step to gather more relevant data at the time of benefit-risk assessment.

To contribute solutions to these recommendations, the IRDiRC Executive Committee set up a Task Force that brought together 35 experts from Europe, the United States and Japan; it met in London on 3rd May 2016. While the Task Force had a strong base of statistical expertise, other contributions including patients, physicians, regulators, industry members, scientists and academics all contributed substantially to the recommendations of the group.

This talk will summarise the main outcomes from the workshop under six headings: different study methods/designs vs. types of conditions; adequate safety data; multi-arm or “platform trial” designs; decision-analytic approaches and rational approaches to adjusting required levels of evidence; extrapolation problems and opportunities; and patients’ engagement in study design.

A report of the Task Force (including a summary of the workshop) is currently in preparation and will be published on the IRDiRC website.

**Acknowledgements**

This abstract is presented on behalf of the IRDiRC SPCT Task Force.

## O8 HAE patient identification and diagnosis: An innovative, ‘game changing’ collaboration

### Thomas J. Croce Jr. (tcroce@shire.com)

#### Head of Global Patient Advocacy, Shire plc, Dublin, Ireland

**Background**

HAE International (HAEi) and the HAE Association (HAEA), which represent hereditary angioedema (HAE) patient groups around the world, in partnership with Shire, developed the HAE patient identification and diagnosis program in response to speed timely and accurate diagnosis. Patients often deal with lengthy delays because of limited awareness and inadequate information. Health care professionals are also often unaware of the disease and its presentation.

**Materials and methods**

This program addressed these problems by identifying gaps in physician, patient and family member knowledge and developing a core set of materials to educate them. As an autosomal dominant disease, family testing was identified by patient groups and the company as having the greatest impact on more timely and accurate diagnosis. General materials were created for all three key stakeholders, providing information on the disease, diagnosis, and confirmatory testing. Additional resources on family testing were created to aid physicians in their discussion with patients, as well as for patients in their discussion with their families. Materials were developed by patients for patients, showing a sensitive approach to their content.

Next, member organisations of HAEi and HAEA were engaged to understand how to use these new materials in their countries. Forty-one member groups worldwide were trained by these larger organisations. HAEi, HAEA and Shire organised webinars regional workshops to facilitate education in countries where there were no patient organisations.

**Results**

This program has resulted in increased awareness of HAE and improved diagnostic training for healthcare professionals internationally. The campaign reached over 340 patients in the United States alone with diagnosis/treatment information. There was very positive feedback from the HAE community with an increase in the number of patients and members of country organisations, including over 700 new members in the United States. Working together, HAEi, HAEA, and Shire have broadened awareness of the disease and increased time to diagnosis.

**Conclusions**

By working together strategically, all parties were each able to bring individual strengths and knowledge to the project for the benefit of patients. The result was not just more information but also a framework for global and regional organisations to ensure that member groups are able to execute effectively. This cooperation between industry and advocates provides an approach that can be replicated across other disease areas.

## O9 Co-creating with the community: primary packaging & administration for people with haemophilia

### Jonas Fransson, Philip Wood

#### Sobi – Swedish Orphan Biovitrum AB (publ.), Stockholm, Sweden

##### **Correspondence:** Jonas Fransson (jonas@fransson.sobi.com) – Sobi – Swedish Orphan Biovitrum AB (publ.), Stockholm, Sweden

Before filing the European marketing authorisation application for Elocta, Sobi wanted to explore whether more user-friendly packaging and administration could make life easier for people with haemophilia. Haemophilia is a life-long, chronic condition that – untreated or undertreated – causes bleeding, including into joints, resulting in progressive joint damage.

Sobi had heard that packaging and dosing syringes are not always designed to meet the needs of people with haemophilia, being sometimes bulky and with syringes that are difficult to manage. This could impact drug administration and treatment compliance, particularly in children learning to self-inject, as well as adults with impaired joint mobility, which can be a consequence of haemophilia.

Working with organisations representing people with haemophilia, Sobi initiated a dialogue with more than 100 individuals, including carers and healthcare professionals. We asked people with haemophilia to record video diaries of their experience of treatment; and travelled to meet with them for open conversations about the topics that they had raised.

The insights allowed us to create a briefing document, which we gave to an award-winning design company. The resulting series of potential new designs for the packaging and syringes were shared with the haemophilia community to explore if these designs would better meet the needs of people with haemophilia.

In a process of ongoing dialogue and constant refinement, we learned that size was the most important element. The resulting packaging is more compact than most conventional therapies, which simplifies storage and travel. It is also recyclable.

We also learned that it is important to facilitate injections, because people might have impaired grip function due to joint damage; and for younger patients with small hands. With the help of the community, we developed an innovative syringe plunger rod with horizontal ribs and an extra centre grip with a soft top, which increases friction and gives equal grip, even if it is not pushed exactly squarely.

The packaging is also complete: everything needed to prepare and inject the treatment is provided, i.e., drug, pre-filled solvent syringe, low-resistance infusion set, sterile swabs, adhesive bandage and a packaging leaflet.

Through genuine co-creation with people with haemophilia and the community, we believe that Sobi has gathered new insights into how packaging can be developed. We were able to build a genuine dialogue with people with haemophilia, their carers and healthcare professionals, which has created opportunities for continued validation and refinement of future approaches.

## O10 Go with Gaucher, taking forward the next generation. How to involve young people to create a new generation of patient advocates

### Anne-Grethe Lauridsen, Joanne Higgs, Vesna Stojmirova Aleksovska

#### European Gaucher Alliance (EGA), Dursley, Gloucestershire, GL11 4ND, UK

##### **Correspondence:** Anne-Grethe Lauridsen (anne-grethe@eurogaucher.org) – European Gaucher Alliance (EGA), Dursley, Gloucestershire, GL11 4ND, UK

**Background**

This event developed the aim: to encourage young adult Gaucher patients to come together to exchange information and ideas; to help improve the quality of life of the participants; to broaden their horizons; to encourage them to take an active role in their national associations and at a European/international level; to support the work of finding and strengthening the future leaders of the EGA and its member associations.

**Content**

The EGA wanted this to be a project that involved all member associations, so once the project plan was created, members were then asked to identify one participant from their own association. The first meeting took place in Frankfurt, November 2012 and was attended by 31 young Gaucher patients, ranging in age from 17 to 34 years, representing twenty of our member countries, mainly from Europe. The main topics: 1/Getting to know each other: This was the first time that most of the group had met and so a lot of time was spent at the beginning of the meeting to create a safe environment where everyone could speak freely. 2/ Learning about Gaucher disease in order to educate the young patients in relation to the management of their condition. 3/Workshops on special subjects that they wanted to discuss. 4/Information about the EGA and the national associations. A second meeting took place in November 2015. There were both new participants and some from the first meeting. The program this time was similar to the first one but also included training sessions on fundraising, social media and public relations.

**Results**

Both participants and member associations highlight that meeting peers, sharing feelings and problems is the most important outcome. Some participants wanted to be more active, but due to current personal situation, i.e. studies, this was not the time. Still many of these young patients have become more involved in the work of their national groups, in EGA activities and even involved in EURORDIS activities. The group has continued to stay in touch using a closed Facebook group. Feedback from the weekend has been used to shape some of the EGA’s work programme: International Gaucher Day, training and raising awareness for doctors, sharing best practices in daily life and developing websites for all national associations.

**Conclusions**

The project Go with Gaucher have reached its goals and have become an ongoing project within the EGA working programme.

## P1 ODAK – Orphan Drug for Acanthamoeba Keratitis

### Christina Olsen^1^, Ritchie Head^1^, Antonio Asero^2^, Vincenzo Papa^2^, Christa van Kan^3^, Loic Favennec^4^, Silvana Venturella^5^, Michela Salvador^5^, Alan Krol^6^

#### ^1^Ceratium Ltd, Merseyside, CH48 8AP, UK; ^2^SIFI SpA, Catania, Italy; ^3^PSR Group B.V. Hoofdorf, 2132HN, Netherlands; ^4^University of Rouen, Rouen, France; ^5^RTC Pomezia 00040 Italy; ^6^Moorfields Pharmaceuticals London L17NH UK

##### **Correspondence:** Christina Olsen (Christina.olsen@ceratium.eu) – Ceratium Ltd, Merseyside, CH48 8AP, UK

Acanthamoeba Keratitis (AK) is a rare infectious eye disease prevalent in contact lens wearers (over 85 % of AK cases) resulting from exposure to *Acanthamoeba* spp., ubiquitous free living microorganisms. Symptoms can include severe eye pain, light sensitivity, vision loss and in extreme cases corneal transplant and enucleation (eye removal). There is currently no licensed drug to treat AK.

The ODAK consortium of industry and academia are undertaking the clinical development of PHMB (Polihexanide). The aim is to apply for marketing authorisations to provide the first approved ophthalmic intervention for the safe and effective treatment of this rare disease. The ODAK consortium have already completed a retrospective study which has shown efficacy of PHMB 0.02 % alone or in combination with other anti-amoebic drugs [1]. A range of non-clinical studies (including pharmacodynamics, pharmacokinetics and toxicology) on novel formulations of PHMB have been carried out and resulting *in vitro* and *in vivo* efficacy tests showed 0.02 % PHMB as the least effective formulation against *Acanthamoeba polyphaga*, with respect to other tested PHMB concentrations (0.04 %, 0.06 %, 0.08 %) [3]. The safety ocular profiles of three PHMB formulations (0.08 %, 0.25 % and 0.8 %) were assessed in an animal model indicating no relevant treatment-related effects of PHMB ophthalmic solutions at 0.08 % and 0.25 %. Only 0.8 % PHMB eye drops showed moderate/severe ocular toxicity [2]. The toxicological data supported the plan to investigate PHMB 0.04 %, 0.06 %, 0.08 % (and placebo) eye drops in a Phase I clinical trial. The trial held at three clinical centres in the Netherlands and Belgium in 90 healthy volunteers was successfully completed in Q1 2016. A Phase III clinical trial is scheduled to begin Q4 2016.

The ODAK project has faced particular challenges in establishing patient involvement in the drug development pathway due to the nature of the disease. Infection is unpredictable, disease outcomes vastly differ patient to patient and engaging patients once disease progression is resolved is difficult. There is no established patient group for AK or patient registry. Patient centric information is lacking and patients can feel isolated, without a forum to translate their experience. ODAK is working with patients to address these issues, identifying and engaging with patients for protocol and patient information leaflet design, developing information on preventative care and support materials for the newly diagnosed, and using the Project website as a go to site for patient stories and advice. The research leading to these results has received funding through the European Community's Seventh Framework Programme under grant agreement No. 305661-2.

**References**

[1] Investigative Ophthalmology & Visual Science April 2014, Vol.55, 5520. Orphan Drug for Acanthamoeba Keratitis (ODAK) project: results of a 10-Year retrospective study in Italy and in the United Kingdom: Vincenzo Papa; Paolo Rama; Federica Ferrario; Andrea Ramoni; Stanislav Matuska; Cherry Radford; John Kenneth George Dart.

[3] Invest. Ophthalmol. Vis. Sci..2015; 56(7 ):1890. The efficacy of Polihexanide (PHMB) eye drops against Acanthamoeba polyphaga investigated by an ATP-bioluminescence assay and a rat model of keratitis. Asero Antonino1; Sudano Roccaro Andrea1; Favennec Loic2; Gueudry Julie2; Le Goff Laetitia2; Blanco Anna Rita1. 1. SIFI SpA (Italy); 2. EA 3800 Universitè de Rouen, Rouen (France).

[2] Date presented at ARVO(The Association for Research in Vision and Ophthalmology) Conference 2016. Poster N. 5395. Ocular tolerability assessment of PHMB (Polyhexanide) 0.8 %, 0.25 % and 0.08 % ophthalmic solutions in rabbits; Asero, Antonino1; Salvador, Michela2; Nyska, Abraham2; Venturella, Silvana2; Papa, Vincenzo1; Blanco, Anna Rita1. 1. SIFI SpA (Italy); 2. RTC, Pomezia (Rome), Italy.

## P5 Rare Navigators help people living with rare diseases to manage the social – and healthcare systems

### Stephanie J. Nielsen, Birthe B. Holm

#### Rare Diseases Denmark, Blekinge Boulevard 2, Høje Taastrup, 2630, Denmark

##### **Correspondence:** Stephanie J. Nielson (sjn@sjaeldnediagnoser.dk) – Rare Diseases Denmark, Blekinge Boulevard 2, Høje Taastrup, 2630, Denmark

Because of the complexity and rareness, people living with rare diseases (PLWRD) are particularly vulnerable in the social- and healthcare systems because standard procedures do not function for these patients. Increased standardization in relation to social support and healthcare procedures jeopardizes the possibility to get the tailored treatment that PLWRD are dependent on, to live good lives.

Rare Diseases Denmark’s Patient Navigators offers structured support to fill in the gaps and prevent PLWRD from falling into cracks. The goal is to equalize disparities and promote health and health literacy in the most vulnerable PLWRDs in Denmark. Based on experience-based knowledge, Navigators provide personal guidance to vulnerable PLWRD to navigate the Danish health care system and to access the social system. The volunteer Navigator either suffers from a rare disease themselves or are a relative to a PLWRD.

Navigators support and empower the wishes, priorities and decisions of vulnerable PLWRD through the principle of *helping them to help themselves*. In addition to the personal knowledge of the Navigators, Rare Diseases Denmark provides an education and continuously support program for the Navigators. Navigators support PLWRD for maximum 12 months - the navigation depends entirely on the individual’s needs and the complexity of the situation.

Providing coordination between and within the systems is a task for the social- and health care professionals. With this project professionals and volunteers combine forces in co-creation contributing to improve the conditions for PLWRD. Through co-creation partners exchange ideas, resources and skills and create new learning environments.

To study the impact of receiving support from a Navigator, all PLWRDs involved in the project, answer the WHO-Five Well-being Index questionnaire in the beginning and in the end of navigation. The results gives an indication of the effect.

Originally the Patient Navigation concept was developed in the US to eliminate barriers to timely diagnosis and treatment of cancer and other chronic diseases. The nation’s first Patient Navigation program was conceived and initiated in 1990 in Harlem, New York, by Dr. Harold Freeman [http://patientnavigatortraining.org/website/documents/prework_%20freeman_principles_of_pn.pdf] Rare Diseases Denmark has adjusted the model and made it suitable for PLWRD.

**Acknowledgement**

Thanks to the national Centers of Expertise for Rare Diseases for cooperation, The Danish Health Authority to grant the project and The Danish Cancer Society for invaluable feedback.

## P6 The eAcademy for Tay-Sachs & Sandhoff disease app

### Daniel Lewi, Patricia Durão

#### The Cure & Action for Tay-Sachs (CATS) Foundation, London SE12 0RW, UK

##### **Correspondence:** Daniel Lewi (dan@cats-foundation.org) – The Cure & Action for Tay-Sachs (CATS) Foundation, London SE12 0RW, UK

Technology has been at the forefront of healthcare and with the increase in dependence on smartphones The CATS Foundation wanted to take advantage of the potential of this media by developing an app using video media that could provide support and information to carers of children affected by Tay-Sachs or Sandhoff.

The charity won the prestigious Patient Advocacy Leadership Award that is run by Genzyme Corporation to create the app. Interviews with families affected by the diseases on subjects ranging from symptom management to advice on care were recorded to provide the ultimate in peer-to-peer support. The app was launched to coincide with the International Rare Disease day in 2015 where it was featured in the national press and publicised through social media.

With an objective of educating carers and parents the app was given the title of the “eAcademy for Tay-Sachs and Sandhoff Disease”. The app was launched across simultaneous mobile phone and tablet operating systems (Android, IOS and Windows) which meant that people would be able to access and download it to their devices.

The majority of downloads have been from people in European countries (47 %) and those in the US (39 %). As the app was developed by a UK based charity and is in English, it is no surprise that the majority of downloads are from people in English speaking countries (71 %).

The development and launch of The eAcademy for Tay-Sachs and Sandhoff Disease app has shown that parents and carers place a high value on our peer-to-peer support model. Users of the app have been able to gain advice on various care related subjects that can have a dramatic affect on their ability to provide high quality care to their children.

As a rare disease, which is notoriously difficult to diagnose, and which has a prevalence rate of only 1 in 320,000 births it was never expected that the app would have a huge download rate. However, the volume of downloads exceeds the total members of The CATS Foundation and has led to families in other countries reaching out to the charity for support and information.

We believe that the app should form a framework for other disease specific organisations. Hearing directly from families about how they manage symptoms and other aspects of care can have a positive impact on users and can lead to them providing a higher level of care for their child.

## P10 The role of a patient organisation in driving the research agenda in a rare disease

### Heather Band, Andrea West

#### Batten Disease Family Association (BDFA), The Old Library, 4 Boundary Road, Farnborough, GU14 6SF, UK

##### **Correspondence:** Heather Band (heatherband@bdfa-uk.org.uk) – Batten Disease Family Association (BDFA), The Old Library, 4 Boundary Road, Farnborough, GU14 6SF, UK

**Background**

Neuronal Ceroid Lipofuscinoses (NCLs), commonly known as Batten disease, are the most common of the rare neurodegenerative disorders of children, affecting approximately 150-200 in the UK. There are currently no cures for this group of devastating genetic diseases. Symptoms include progressive dementia, motor decline, visual failure, and complex epilepsy leading to death at an early age.

**BDFA Research Strategy**

Research funding for rare diseases is fragmented and inadequate. Patient organisations play a vital role in driving the research agenda; to understand the disease mechanisms and bridge the gap from bench to beside. The BDFA has developed a focused and innovative research strategy meeting the complex demands of all 14 types of the NCLs. Key strategic aims and collaborations have been identified to promote excellence in the field of NCL research.

The development of a screening method, using zebrafish, in CLN2 disease, by Dr. Claire Russell at the Royal Veterinary College, London has identified a potential lead compound. Key to this project, was the inclusion of direct family involvement and continuity of project funding to ensure progress. Employing a robust, but flexible funding model allowed for the identification of a potential repurpose use for a compound in CLN2 disease and the establishment of an important research resource for future studies.

The long term funding of a PhD studentship (Sophia Kleine Holthaus) Institute of Ophthalmology (UCL) completed the challenging first steps in finding a gene therapy treatment for visual failure in Batten disease.

**Conclusions**

The current BDFA research strategy produces excellent results. This innovative approach makes considerable and quicker progress towards identifying potential therapies. The key role of a patient organisation in supporting investigators and driving research should not be underestimated and the one key area to address is facilitating dialogue with key stakeholders at an even earlier stage, most notably raising the profile of the research with potential commercial partners and the regulatory authorities. For rare diseases, driving a research agenda makes a faster route to successful therapies more likely. Innovation is key to where resources are limited.

Carefully placed funding can produce high quality work enabling researchers to apply for significant research awards from major grant funding bodies. Projects funded by the BDFA were integral to successful award to the BATCure consortium of a 3-year Horizon2020 research grant.

**Acknowledgements**

BATCure consortium is funded under the EU Horizon2020 research innovation programme under grant agreement 666918. Project Coordinator Professor Sara Mole (UCL).

Institute of Ophthalmology (UCL) PhD studentship Professor Robin Ali, Professor Sara Mole (UCL).

Dr. Claire Russell, Royal Veterinary College, CLN2 project supported by Karen and Martin Freeman.

## P13 Expertise for rare diseases mapped

### Marinda J.A. Hammann, Marije C. Effing-Boele, Hanka K. Dekker

#### Patient Organisation for Metabolic Diseases (VKS), 8031 GJ Zwolle, The Netherlands

##### **Correspondence:** Marinda J.A Hammann (info@expertiseinkaart.nl) – Patient Organisation for Metabolic Diseases (VKS), 8031 GJ Zwolle, The Netherlands

**Background**

Patients with rare diseases often have to endure a late diagnosis, a lack of knowledge among specialists and healthcare workers, a lack of effective treatments, and insufficient attention to scientific research. To provide proper care, it is important to cluster expertise for these diseases. This insight has led to the formation of centres of expertise and also leads to the establishment of European Reference Networks. In the process of identifying and designating expertise in rare diseases at national level, hospitals and healthcare workers generally indicate their expertise. The perception of patients is often not incorporated. The goal of the project ‘Expertise Mapped’ is to visualise the organisation of care for patients with rare diseases, from patients’ perspective.

**Method**

**Fig. 1 (abstract P13).**
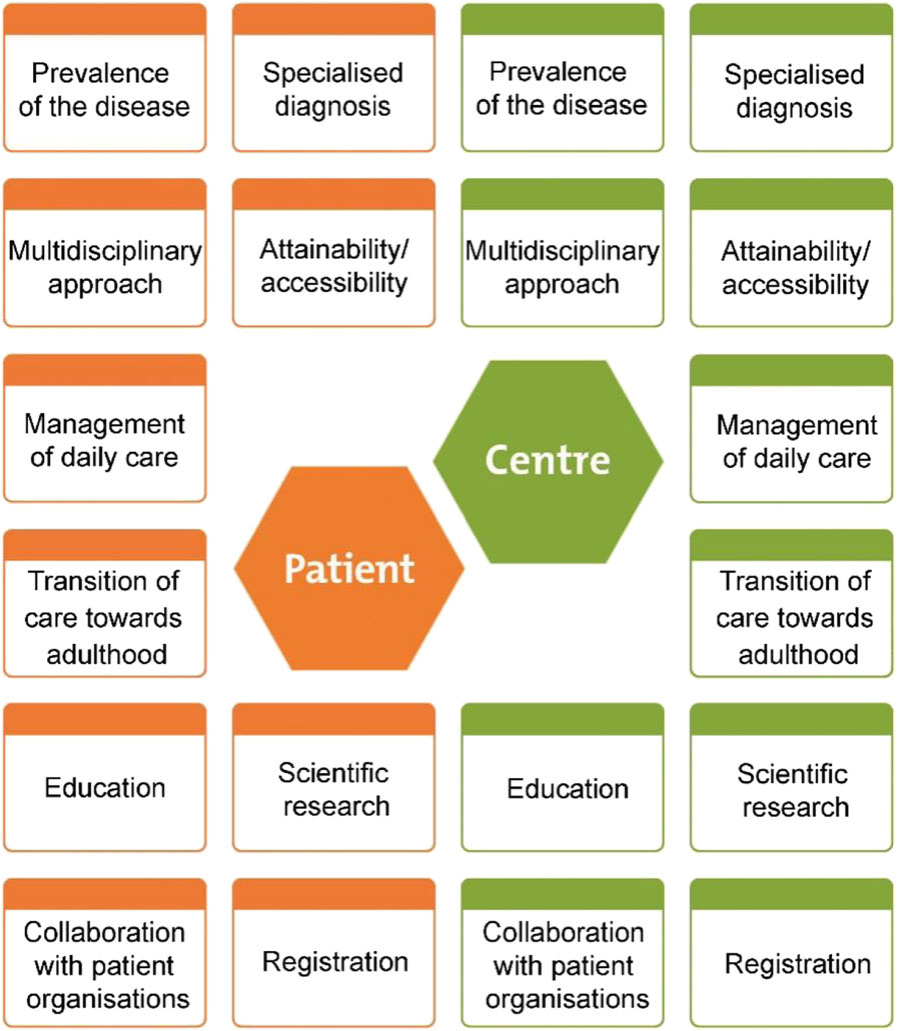
Example of a knowledgemap

The knowledge maps are based on the EUCERD Quality Criteria, summarized in ten topics (Fig. 1). These maps show on the left side the perceptions, needs and wishes of patients (gathered by online surveys) and on the right side the competences of the centre (gathered from visiting the experts by a panel of patients). If there are more centres of expertise for one rare disease, each centre will get their own knowledge map.

**Results**

We produced knowledge maps for 15 different rare diseases, corresponding 27 centres of expertise in the Netherlands. In the next 3 years we will continue producing knowledge maps with the intention to generate a large and diverse overview of rare diseases and their centres of expertise. They are published on the website Expertise Mapped [http://www.expertisemapped.org].

**Conclusions**

It is important to acknowledge centres of expertise for rare diseases from patients’ perspective and to visualise the organisation of care. ‘Expertise Mapped’ provides patients, patient organisations and healthcare workers more insight in the organisation of care, and an overview of the expertise for rare diseases from patients’ perspective. Knowledge maps have many advantages:

1. Knowledge maps show where expertise is located and how healthcare is organised;
2. If there are more centres for the same disease, knowledge maps give patients the opportunity to choose what fits their needs;
3. The online survey provides insight into patients’ perspective on proper care and their actual care needs;
4. Knowledge maps give centres of expertise the opportunity to show their expertise.

‘Expertise Mapped’ strengthens the position of patients by bundling and showing the expertise for different rare diseases from the patients’ perspective. The more knowledge maps are published, the stronger the patients’ voice.

**Acknowledgements**

The project ‘Expertise Mapped’ is a collaboration between different patient organisations for rare diseases and it is funded by the Dutch Government (FondsPGO). The Patient Organisation for Metabolic Diseases (VKS) is project leader. The project partners for 2016 are the Patient Organisation for Pulmonary Fibrosis, the Vasculitis Foundation, the Pulmonary Hypertension Foundation, the Patient Organisation for Aplastic Anemia & Paroxysmal Nocturnal Hemoglobinuria, Patient Organisation for Adrenal Gland Diseases (BijnierNET), the Patient Organisation for Anorectal Malformation and the Organisation for Tall People.

## P14 The hidden costs of rare diseases: a feasibility study

### Amy Hunter, Amy Simpson

####  Genetic Alliance UK, London, N1 3QP, UK

##### **Correspondence:** Amy Simpson (amy.simpson@geneticalliance.org.uk) – Genetic Alliance UK, London, N1 3QP, UK

**Objective**

Genetic Alliance UK are undertaking the ‘Hidden Costs’ project, to test the feasibility of researching the psychosocial and economic costs of models of health service delivery for rare diseases, from the perspective of patients, their families and the NHS in the UK.

**Method**

The feasibility study, overseen by a steering group, focuses on 6 rare conditions and undiagnosed conditions. The study includes a literature review, exploratory meetings with patient organisations, semi-structured interviews (with patients, carers, healthcare professionals and commissioners) and an assessment of approaches to data collection.

**Results**

The literature review has demonstrated that little systematic research has been conducted in this area, and our understanding of the true costs and the impact of the way health services are delivered for rare conditions is limited. Emerging findings confirm that health services for rare conditions are organised in many different ways, with patients and families experiencing varying levels or types of care coordination depending on whether they have a diagnosis, which condition they have, their age, where they live and which service they choose to access. Qualitative interviews are revealing a range of different and ‘hidden’ costs that patients and families face as a result of managing their condition and coordinating their care. Costs include financial costs, such as those associated with travel to frequent appointments, and significant psychosocial costs - for patients, parents (of affected children) and wider family members. A huge cost faced by patients and their parents was time – the time individuals spent ‘project managing’ their care was described as a significant burden.

**Conclusions**

Managing diagnosed and undiagnosed rare conditions can be stressful and costly, for both families and the NHS. Understanding the full costs, both economic and psychosocial, of managing rare disease patients through different models of service coordination will be valuable evidence for decision makers and commissioners in the NHS as they plan service developments and support best practice. A final report detailing the results of the feasibility study will be produced in 2016 to inform future research proposals in this area.

**Acknowledgements**

The Hidden Costs project has been undertaken by Genetic Alliance UK, funded by Shire through a Services Agreement and by Genzyme through a restricted educational grant. Shire’s support made the project possible. Genzyme’s funding enabled us to study more conditions and in greater depth.

## P15 FDA’s new natural history grant program: support to build a solid foundation for development of products for rare diseases

### Gumei Liu, Katherine Needleman (Katherine.Needleman@fda.hhs.gov), Debra Lewis (debra.lewis@fda.hhs.gov), Gayatri Rao (gayatri.rao@fda.hhs.gov)

#### Office of Orphan Products Development, Office of Special Medical Programs, U.S. Food and Drug Administration, 10903 New Hampshire Avenue, Silver Spring, MD 20993, USA

##### **Correspondence:** Gumei Lui (gumei.liu@fda.hhs.gov) – Office of Orphan Products Development, Office of Special Medical Programs, U.S. Food and Drug Administration, 10903 New Hampshire Avenue, Silver Spring, MD 20993, USA

The Office of Orphan Products Development (OOPD) at the U.S. Food and Drug Administration (FDA) announced the launch of a new grant program to fund natural history studies for rare diseases on February 29, 2016.

Successful product development for rare diseases depends on having a thorough understanding of the disease’s natural history. A natural history study of a rare disease captures the course a disease takes in affected individuals from the time immediately prior to its inception, progressing through a pre-symptomatic phase and different clinical stages, to a final outcome in the absence of treatment. [1] They can be useful for defining appropriate outcome measures, biomarkers and subpopulations.

Information about the natural histories of many rare diseases is often incomplete or lacking due to the scarcity of patients to study and lack of resources/funding. While there are nearly 7,000 known rare diseases, there are only 518 approved orphan drugs and 69 approved humanitarian use exemption devices (as of January 29, 2016). Well-conducted natural history studies are needed to expedite the development, review, and approval of products for the many untreated rare diseases. The new natural history grants program addresses this critical need.

The natural history grants program aims to accelerate the clinical development of products for use in rare diseases or conditions where no current therapy exists or where there is a need for therapy superior to existing therapies. The program supports studies that characterize the natural history of rare diseases/conditions, including identifying genotypic and phenotypic subpopulations, and developing and/or validating clinical outcome measures, biomarkers and/or companion diagnostics. There are two funding levels for this grant program. Prospective natural history studies are eligible to be funded at the maximum of $400,000 in total cost per year for up to five years. Retrospective natural history studies or survey studies are eligible to be funded at the maximum of $150,000 in total cost per year for up to two years. OOPD intends to commit approximately $2 million to the program and award 2-5 grants in Fiscal Year 2017 (October 1, 2016-September 30, 2017). This grant program is open to all stakeholders in the international rare disease community, including patients/patient advocacy groups, clinicians, researchers and industry partners.

**Reference**

1. Posada de la Paz M, Groft S. Rare diseases epidemiology. Dordrecht: Springer; 2010

## P17 Understanding the wider impact of adrenal insufficiency: patient organisation involvement in the TAIN project

### Amy Simpson^1^, Amy Hunter^1^, Martin J Whitaker^2,3^

#### ^1^ Genetic Alliance UK, London, N1 3QP, UK; ^2^ University of Sheffield, Sheffield, S10 2TN, UK; ^3^ Diurnal Limited, Cardiff, CF14 4UJ, UK

##### **Correspondence:** Amy Simpson (amy.simpson@geneticalliance.org.uk) – Genetic Alliance UK, London, N1 3QP, UK

**Background**

Those affected by adrenal insufficiency require daily glucocorticoid hormone replacement therapy. Currently, there is no licensed treatment for patients under 6 years of age in Europe and existing treatment often requires adapting adult doses by crushing tablets. Until now, little was known about the psychosocial impact of living with and treating adrenal insufficiency.

**Objective**

The European Commission funded TAIN project is developing an innovative formulation of hydrocortisone for children affected by adrenal insufficiency. Genetic Alliance UK is working with parents of affected children to ensure that their experiences inform the development of effective treatments.

**Method**

Taking a mixed methods approach, Genetic Alliance UK conducted and analysed 17 semi-structured interviews with parents in the UK. Subsequently, an online survey was developed, piloted and disseminated widely to parents of children under the age of 6 in the UK, the Netherlands and Germany. Detailed analysis is ongoing. The study was approved by the University of Sheffield’s research ethics committee.

**Results**

Findings have provided an insight into the wider impact of adrenal insufficiency, the challenges associated with parenting a child with a rare condition and the chronic treatment regime. Interviewees reported a lack of awareness of the condition, delays in getting an accurate diagnosis and disparities in care across the UK. Although parents reported that their child’s condition was relatively well managed, many described a number of challenges associated with the treatment regime and made suggestions for how it could be improved. They reported disruption to their daily routines, anxiety about administering the right dose at the right time and difficulties in delegating responsibility for their child’s care and medication. The challenges were particularly acute for new parents.

**Conclusions**

Genetic Alliance UK’s involvement has added value to the TAIN project. It has increased understanding of the reality of living with adrenal insufficiency from the family perspective. This context is critical to the development of interventions, which are lacking for many rare diseases. Dissemination of results to families and stakeholders will raise awareness and knowledge of the impact of this rare condition. The research has highlighted unmet needs of families and helped identify areas for future research and opportunities to influence policy of benefit to patients. The TAIN project has laid the foundation for a long-lasting collaboration between patient organisations, industry and academia in the field of adrenal insufficiency and provides a useful example of the potential benefits of such a partnership.

**Acknowledgements**

The TAIN project is funded by the European Commission under a Framework 7 Grant (No: 281654 – TAIN) [www.tain-project.org]

## P20 Bridging the gaps between medical and social care for people living with a rare disease

### Raquel Castro (raquel.castro@eurordis.org)

#### European Organisation for Rare Diseases – EURORDIS, Paris, France

**Background**

People living with a rare disease (RD) and their families have significant health and social challenges which affect their dignity, autonomy and other fundamental human rights. In order to overcome these challenges, people living with a RD often need multidisciplinary health, social and local care and support. However, the various services involved in care provision are commonly managed by different authorities and providers, and there is a lack of communication and coordination between them.

**Materials and methods**

The objective is to address the gaps in the coordination between health, social and local services responsible for supporting people living with a RD and their families, by developing a holistic personalised care pathway.

The innovative care pathway results from 1) multi-stakeholder work on the identification of key issues to support the incorporation of RD into social services, performed within the EUCERD Joint Action for RDs (2012-2015); and 2) literature review on integrated care provision in health care settings and beyond.

**Results**

The innovative care pathway involves linking health services to social and support services that people living with a RD and their families use on a daily basis, ensuring the transfer of information and expertise between service providers. The care pathway also centralises the coordination of care through a resource centre for RD and regional case managers, in an effort to relieve the burden of care management for people living with a RD and their families. This optimised care model is expected to also result in efficiency gains for national authorities.

This innovative care pathway is the core of the new EU-funded project INNOVCare (*Innovative Patient-Centred Approach for Social Care Provision to Complex Conditions*, 2015-2018) and will be implemented in a pilot in Romania. Its socio-economic impact and cost-benefit will be assessed by social innovation and health economics experts.

**Conclusion**

The low prevalence and complexity of RD, as well as the significant unmet needs of RD patients, highlight the need for the implementation of holistic, integrated and patient-centred care pathways, which respond to the RD challenges through an interdisciplinary approach. INNOVCare pilot will be an important step into understanding the game changing potential of integrated care pathways on the daily lives and autonomy of people living with a RD and their families.

**Fig. 2 (abstract P20).**
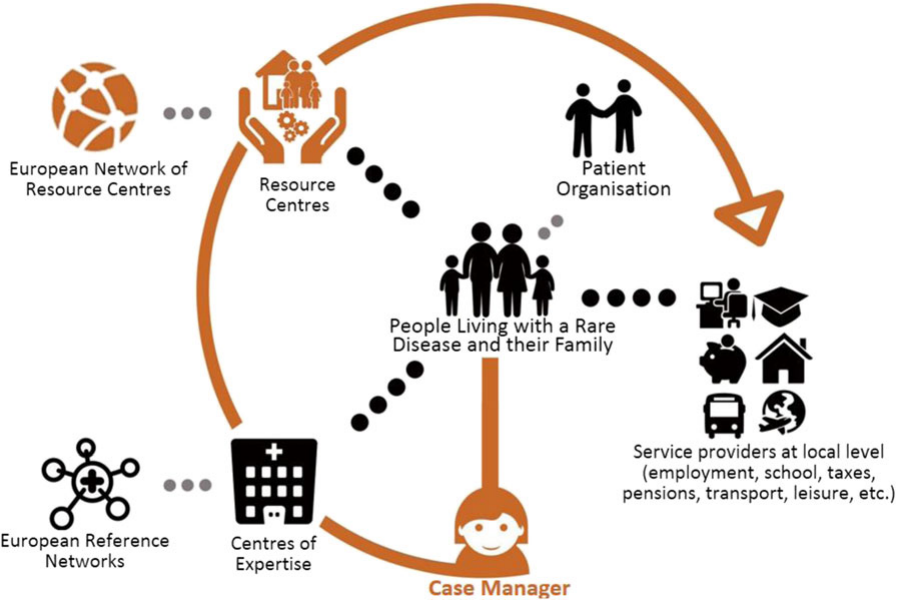
Innovative patient-centred care pathway proposed by the INNOVCare project

